# Australian nurses in general practice, enabling the provision of cervical screening and well women’s health care services: a qualitative study

**DOI:** 10.1186/1472-6955-11-23

**Published:** 2012-11-12

**Authors:** Jane Mills, Jennifer Chamberlain-Salaun, Leane Christie, Margot Kingston, Elise Gorman, Caroline Harvey

**Affiliations:** 1 School of Nursing, Midwifery & Nutrition, James Cook University, Cairns Campus, Cairns, QLD, 4878, Australia; 2 Queensland Cervical Screening Program, Queensland Health, Herston, QLD, 4029, Australia; 3 Sexual Health & HIV Service, Queensland Health, Brisbane, QLD, 4000, Australia; 4 General Practice Queensland, Brisbane, QLD, 4001, Australia; 5 Family Planning Queensland, 100 Alfred Street, Fortitude Valley, QLD, 4006, Australia

## Abstract

**Background:**

The role of Australian general practice nurses (PNs) has developed exponentially since the introduction of service based funding in 2005. In particular, their role has expanded to include cervical screening and well women’s health care services provided under the supervision of a general practitioner (GP). While previous research identifies barriers to the provision of these services, this study sought to investigate enablers for nurse led care in this area.

**Methods:**

A number of grounded theory methods including constantly comparing data, concurrent data collection and analysis and theoretical sampling are utilised in this qualitative, exploratory study. A purposive sample of PNs who completed the required program of education in order to provide cervical screening and well women’s health care services was recruited to the study. Data is presented in categories, however a limitation of the study is that a fully integrated grounded theory was unable to be produced due to sampling constraints.

**Results:**

Four enablers for the implementation of a change in the PN role to include cervical screening and well women’s health checks are identified in this study. These enablers are: GPs being willing to relinquish the role of cervical screener and well women’s health service provider; PNs being willing to expand their role to include cervical screening and well women’s health services; clients preferring a female practice nurse to meet their cervical screening and well women’s health needs; and the presence of a culture that fosters interprofessional teamwork. Seven strategies for successfully implementing change from the perspective of PNs are also constructed from the data. This study additionally highlights the lack of feedback on smear quality provided to PNs cervical screeners and well women’s health service providers.

**Conclusions:**

The influence of consumers on the landscape of primary care service delivery in Australia is of particular note in this study. Developing interprofessional teams that maximise each health care provider’s role will be fundamental to comprehensive service delivery in the future.

## Background

The research question this qualitative study seeks to address is: What are the enablers and barriers for PNs translating knowledge acquired from a training program for the provision of cervical screening and associated well women’s health care services into practice? The aim of the study is to examine the process of changing the traditional division of labour related to cervical screening and well women’s health care services in the general practice setting.

While previous studies have examined the role of PNs as Pap smear [cervical screening] providers [1-4] findings are limited to identifying barriers to implementing the role, while recommending further research in the area. This study builds on previous work in order to develop a solutions based approach for PNs and their employing general practices wishing to incorporate cervical screening and well women’s health care services into their work. Findings from this study are largely framed in terms of enablers for change in the context of general practice, and include the influence of workplace culture, educational opportunities and communication strategies on the implementation of a new model of nurse led care. Strategies employed by both the participants and their colleagues to overcome previously identified barriers to changing models of practice nursing to include cervical screening and well women’s health care services were identified and further recommendations made based on these findings.

The role of Australian PNs has expanded rapidly over the last decade with PNs increasingly providing cervical screening and well women’s health care services during this period. In 2005, this role expansion was recognised and encouraged by the addition of item numbers to the Medicare Benefits Schedule (MBS) for PN consultations and the development of business cases to justify financial viability. The MBS item numbers for cervical screening and preventative well women’s health checks were 10994, 10995, 10998 and 10999 with the explanatory notes for these item numbers stating that:

The practice nurse must be appropriately qualified and trained to take cervical smears and other preventive checks. This means that where credentialing arrangements are in place, the practice nurse should be credentialed as qualified and trained to take Pap smears. All practice nurses taking Pap smears and other preventive checks should have undertaken an accredited training course."

*Continuing professional development is a compulsory part of the credentialing arrangements and is recommended for all nurses taking Pap smears and providing preventive checks in jurisdictions where there are currently no credentialing arrangements*[5].

While the Practice Incentive Program (PIP) Cervical Screening Incentive [6] has provided a financial incentive to GPs to encourage the screening of under-screened women (women who have not had a Pap smear in 4 years) for cervical cancer and to increase overall screening rates, on the 31^st^ of December 2011, MBS PN item numbers covering immunisation (10993), cervical smears (10994, 10995, 10998 and 10999) and the treatment of a person’s wound (10996) were made redundant. As of the 1^st^ of January 2012 the Practice Nurse Incentive Program (PNIP) consolidates previous funding arrangements for these six MBS item numbers and the Practice Incentive Program (PIP) Practice Nurse Incentive. Funding under the PNIP provides eligible general practices, Aboriginal Medical Services and Aboriginal Community Controlled Health Services incentive payments to support an expanded and enhanced role for PNs working in these organisations [7] that is no longer reliant on billing for specific tasks.

In Australia, different jurisdictions have regulated the practice of nurses providing cervical screening services in response to legislative requirements and professional practice approaches. The state of Victoria was an early adopter of a credentialing system and subsequent access to cervical screening pathology services for nurse ‘Pap smear’ providers. This model allows the collection of data demonstrating the increase in nurse provided cervical screening and is also valuable in measuring the quality of nurse provided cervical screens [8].

Australian States and territories have followed similar pathways in terms of educational preparation and authorisation. In the context of this study Family Planning Queensland (FPQ) provides a *Pap Smear Provider Module* incorporating a three-day workshop and clinical attachment completed within six months of finishing the theory component. The Royal College of Nursing Australia accredits the FPQ module. In Queensland, nurses who successfully complete this program of education can apply for authorisation with Queensland Health as an authorised non-medical cervical screening provider; this authorisation allows the nurse to access the Queensland Health Pap Smear Register to obtain details of women’s past cervical screening history. Authorisation in Queensland however, does not extend to nurses being able to generate requests for pathology services, as is the case in Victoria and Western Australia. Authorisation as a non-medical cervical screening provider is renewed every three years and requires nurses to submit documented evidence of ongoing competency.

## Methods

A qualitative research design utilising individual interviews and grounded theory methods of analysis was deployed to answer the research question posed. Grounded theory methods [9] are useful in researching phenomena about which little is known. Grounded theory methods of constantly comparing data and concurrent data collection and analysis were used to explain the area of interest in this study. These methods prioritise processes used by participants to manage a particular phenomenon in their everyday lives [10], which was a suitable approach for this study.

The study population is registered nurses working in general practice enrolled in the *Pap Smear Provider Module* facilitated by FPQ between January 2005 and March 2011. A purposive sample of PNs who believe they have successfully implemented a change in their clinical work to include the provision of cervical screening and related well women’s health checks was recruited by FPQ and General Practice Queensland (GPQ) with a final sample size of 18 PNs.

Participants were interviewed about their experience of introducing a change to the traditional model of service delivery in their general practice workplace using a semi-structured guide that was amended after the analysis of each interview in order to refine the focus of data to be collected subsequently. At the beginning of each interview a grand tour question “Tell me about your experience of providing cervical screening and well women’s health checks in your practice?” was asked of each participant. Interviews were transcribed and analysed using both open and focused codes related to the enablers for change.

This study was granted ethics approval from the James Cook University Human Research Ethics Committee (H3847). Written consent was obtained from all participants prior to interviews being undertaken.

## Results

Participants perceive four key enablers to implementing a model of nursing care that included the provision of cervical screening and well women’s health services. The four enablers are: GPs being willing to relinquish the role of cervical screener and well women’s health service provider; PNs being willing to expand their role to include cervical screening and well women’s health services; clients preferring a female practice nurse to meet their cervical screening and well women’s health needs; and the presence of a culture that fosters interprofessional teamwork. Importantly the process of enacting each of these enablers is conditional on all members of the general practice team being in a position to identify their own professional development needs as well as gaps in service delivery related to their particular practice.

### General practitioners relinquishing a traditional role

Participants identify that overall the GPs they work with are supportive of their role as well women’s health care providers. The reason why some GPs relinquish the provision of cervical screening in particular, and well women’s health services more generally is multifactorial and varies from general practice to general practice and within individual general practices.

Two participants perceive the ethnic backgrounds of some of the male GPs in their practices as influential in their decision to delegate this role to a PN. “One of them [male GPs] is a Sikh, so his religion sort of, you know, basically it’s easier for him to have a female nurse do them for him” (M10r119). Other participants thought that some GPs lack confidence in providing the service with one participant stating, “I think they [male GPs] felt a little uncomfortable doing the Pap smears themselves” (M16r31). Another commented that the procedure is “a little bit frightening for them, because they’re not females themselves” (M7r119). This same participant also comments that during a conversation with GPs she asked if they had done “obs and gynae” during their training and was told “No, we’re just expected to know how to do it” (M7r285).

“The men [male GPs], they couldn’t wait to offload them (M9r70)…I don’t think a lot of male GPs like doing Pap smears from what I’ve found, and it’s something that they are quite happy to get rid of”. (M9r74)"

One participant in the study identifies that while the male GPs in the practice where she works are still committed to providing cervical screening and well women’s health checks, the influence of female GPs being willing and happy to relinquish this role had resulted in her being authorised as a provider of cervical screening services.

“Two of the doctors [male GPs] flat out refused to let anyone else but themselves do Pap smears..[however] a lot of the female patients particularly asked for a female doctor to do their Pap smears and so [the female GPs] were always getting the [male GPs’ Pap smear clients] dumped on them…and they got sick of, you know, just seeing females…they wanted a bit more out of their practice…so they thought well a nurse could do them”. (M10 r71)"

### Practice nurses expanding their role

For many PNs, developing knowledge and skills that can contribute to the expansion of their role in general practice is important, however as one participant pointed out it is equally important to acknowledge and “accept that there’s a lot of nurses that don’t want any more responsibility, or they don’t want to extend their roles. They’re happy to work within…what they currently do” (M3r218). For some participants their interest in women’s health and the needs of clients are motivating factors for them expanding their roles to include the provision of cervical screening, as the following quotes demonstrate:

“I’m a midwife and I see lots of women ante-natally and in general practice and one of the common things that I kept on having women say to me [was] ‘I feel comfortable with you. Why can’t you do a Pap smear with my six week post natal check?’ and so I thought…I understand why women would like familiarity with somebody…and so I sort of felt as though it would be something that I can add value to my service to women”. (M17r11)."

While electing to undertake a course in preparation for providing women’s health services is ultimately an individual PN’s choice, findings indicate that in some instances it may be a practice manager, a practice principle or a division of general practice who identifies a need and initially suggests that a PN undertake the training. “One of our lady doctors heard that they [Division of General Practice] had sponsorship funding available and suggested that I do it”. (M10r31).

Sharing knowledge with clients is an educational opportunity that PNs find rewarding in the reconfiguration of their role to include cervical screening and well women’s health services. PNs use these consultations as an opportunity to provide clients with information regarding sexual health and other health matters. As one participant pointed out, providing a cervical screening service enables her to “catch the young ones that are having sexual intercourse earlier, and just, you know, trying to educate the young kids on HPV [human Papillomavirus]” (M1r23). PNs may also extend the educational aspect of their role beyond the confines of consultation times and organise community health education events. One participant working in a remote area practice explains how she hosted a webcast event for clients and their partners about women “making their forties fabulous”. The event included dinner and encouraged clients to discuss any issues with the PN after the webcast (M4).

### Client preference

Participants in this study identify an important enabler to a new model of service delivery as being the number of clients who prefer a female clinician to provide their cervical screening and well women’s health services. Prior to one participant commencing employment, the general practice where she works had a “list of women who were waiting to have their Pap smear, once either a female doctor started or they had a [female] nurse doing it as well”. (M16r81). Interestingly, another participant also perceives a difference between clients related to age, with younger women much preferring a female clinician.

“The younger girls would rather see a female whether that’s a nurse or a doctor; they would just rather a female…The older ladies who’ve had all their babies…they don’t care. They know it’s got to be done…they probably prefer a lady but they are not going to complain if they have to see a man. But I think the early age group… to them it’s important that they see a lady”. (M9r170)"

Meeting general practice’s female clients’ expressed needs provides the impetus for a number of PNs to enrol in the training program as the following participant, who works in a practice with all male GPs, explained:

“I have started in a new surgery and a lot of women were coming in and wanting to know if there was a female there, you know, mainly to do Paps smears. So I thought, well, I should do something about this”. (M2r39)"

Reasons cited for clients choosing PNs over GPs to provide cervical screening and well women’s health checks include: Difficulty in getting an appointment with a female GP, the familiar face of a PN who works in a practice with high GP turn over, PNs’ listening skills, the additional time that PNs have to spend with clients, and that; clients feel more comfortable with a PN and are more likely to ‘talk’ about their women’s health needs.

“They can chat to me about things they would never dream of talking [about] to a GP. I’ve had women come and say “Look, I’ve never asked a GP about this, but…” you know, and ask me things”. (M4r28)"

An important finding in this study was that a client choosing a PN to provide cervical screening and well women’s health checks indicates their understanding of these procedures as preventative care as opposed to treatment for an identified illness.

“Sixty percent [of clients] at the moment are choosing me. We think a lot of the reason behind this is simply because they’re not unwell. They’re well women and they’ve said, “I don’t need to see the doctor, there’s no reason for seeing the doctor” (M14r215)… They still have that idea [a doctor is for when you are unwell] in their heads, so that’s probably where our primary health care message in nursing is coming a lot stronger. Umm because we’re looking at it saying look, we’re able to provide this preventative health measure and people are still thinking, well, you know the doctor’s for when I’m sick…so that’s where I think nurses in general practice will probably lead the way”. (M14r219)"

In rural and remote areas GPs are often either visiting or change regularly. A PN therfore can be the most consistent healthcare provider in these communities and as such they feel part of the community in a way that GPs may not, with a concomitant sense of responsibility. The same rural PN who organised the webcast event in her community also describes how she views her role as:

“Not just going and learning a training program and bringing that [back] on a nine to five basis, but really expanding that out into the community” (M4r229)….you feel that anything that you can do to help provide better care, or provide information, or just be an avenue where someone might come and need to talk to you. I think it is a really important part, because there’s often not the resources that there are in other places or people aren’t sure where to avail themselves of those resources”. (M4r231)"

### A culture of interprofessional teamwork

Findings from this study demonstrate that narrowing the gap between assessed community need and the services provided by a general practice relies on the development of an interprofessional team demonstrating collegial interactions, shared planning for policy and procedure frameworks, a commitment to appropriate infrastructure, and an investment in knowledge and skills development. Each of these factors influence PN’s ability to provide cervical screening and well women’s health services with the lower end of the scale resulting in PN’s role being relegated to that of a “Pap smear technician” (M14r171, M17r244).

The quality of collegial interactions between PNs, GPs, receptionists and practice managers is an important consideration in changing models of health service provision in general practice. Participants cited many positive collegial interactions with general practice team members using words such as “support”, “mutual respect”, “trust” and “acceptance” to characterise their interprofessional relationships. One participant employed in a private practice with four GPs comments that the GPs “had instant trust to let me go ahead” (M1r133) based on her previous experience of working in the practice and the quality of the course of education she had undertaken.

In practice environments where collegial interactions are limited, PNs are unlikely to provide full cervical screening and well women’s health services and may be limited to providing a technical service; undertaking a Pap smear service as part of a GP consult.

“I do the Pap smear, the doctor would check the cervix, then the consult would continue and I would just leave the room. So I was really there just to do the mechanical thing of taking the Pap smears” (M12r51)."

Receptionists, and in some instances practice managers, are a general practice’s front line contact and as such play a key role in promoting nurse led cervical screening and well women’s health clinics. Delivering a quality service requires the support of the whole practice team in order for a change to be effective. The importance of receptionists to the successful delivery of services is highlighted in the following participant’s comment that “receptionists are the gatekeepers always and if they are not promoting your service you’re never going to get any patients”. (M3r170). Another participant identifies the commitment and support of the practice manager as instrumental in enabling her to deliver a well women’s health clinic as opposed to “having that [Pap smear] technician type role” (M14r171).

PNs working in practices that have developed specific position descriptions incorporating the provision of cervical screening with policies and procedures related to the service are more likely to provide well women’s health checks than practices that do not have this infrastructure in place. Developing documented cervical screening processes provides an opportunity for team members to consider and decide how cervical screening will be delivered in their particular general practice and to allocate tasks and responsibilities. Numerous participants referred to policy frameworks as a positive mechanism for identifying the scope of their practice.

“I think that’s really important to…decide what role I was going to play, how far I was going to take it, let’s say, if I’m doing the Pap smear and was I going to follow up the results, was I going to inform the patients of the results…was all that my responsibility or did they (GPs) just want me to just physically do the Pap smears” (M3r95)"

Space and time, or a commitment to infrastructure, are also important factors in the provision of Pap smears. Dedicated PN consulting rooms and appropriate duration of appointment time allocations enables PNs to run nurse-led clinics, including cervical screening and well women’s clinics. Dedicated consulting space allows ownership of a space that PNs can adapt and arrange to suit their individual practice and provides a comfortable, safe, private environment for clients. Time allows comprehensive client histories to be taken and provides an invaluable opportunity for PNs to identify other health issues that a client may be experiencing and an opportunity to provide health information and referral. In this study, almost half of all participants have access to dedicated space and are allocated between 20 and 45 minutes for this service, with 30 minutes being the most common time allocation. The following participant’s quote demonstrates the significant benefits that dedicated PN space and time accords clients:

"I have one of the consulting rooms…Each month we work out what days I’m doing my clinic and that room…[is] booked out to me (M14r255). I have half an hour appointments with clients and if they [the client] feel that I’m not pressed for time, they’re more willing to sort of [say] “well actually there’s something else bothering me too” (M14r115)."

Developing the required knowledge and skill set to effectively provide a cervical screening and well women’s health check clinic extends beyond PNs attending a course of education. Knowledge exchange with GPs and peers provides a valuable teaching and learning opportunity that develops interprofessional teamwork. The availability and accessibility of GP’s expert advice supports the extension of PN’s knowledge beyond that acquired in the course.

"If I need to talk something through…there is always an open [GP] door there…I see something visually that I mightn’t be sure of, that might be a little bit different…I can grab one of them [GP} to come and have a look (M6r19)."

One participant also spoke of sharing knowledge gained during cervical screening training with a newly trained GP:

"I’ve just had a new one [GP] start…she’s through the medical school…and just starting her GP now, and she said “Look we weren’t educated to this max at all”, and so…I’m in there doing with her, with her Paps teaching her if she’s just, you know, like she’s unsteady (M1r49)."

Peer networks also provide PNs with valuable support and additional opportunities to exchange knowledge and seek advice. Peer networks are usually formed and coordinated through divisions of general practice, although continuity of networks is reliant on individual’s commitment and a coherence between members.

"I had a sixteen year old who…had booked in for it [a Pap smear] and I was thinking, oh sixteen, you know, that’s a bit young. But discussed it with the others and they said “Oh no, it’s sixteen” but then I had to get back to them and say well, she’s been sexually active for four years and she has got a baby already, so you know. And they all came back and said “Oh well, yeah you’ve go to do it in that case”. But it flows outside the criteria of when you are supposed to do Pap smears, so I wasn’t too sure about it, but we got is resolved in the end (M10r279)."

## Discussion

Embedded in the findings of this study are seven strategies that participants used to successfully implement a model of PN led care (Table 1).

**Table 1:** Strategies for successfully implementing a practice nurse led model of care

1	Appraise the local general practice culture
2	Identify goals for role development and expansion that benefit practice nurses as individuals
3	Identify goals for role development and expansion that benefit clients of the general practice
4	Assess patients’ preference for a female clinician to undertake cervical screening and well women’s health services
5	Implement a model of practice nurse led care that is appropriately resourced
6	Foster interprofessional communication and collaboration within the team in order to meet patient’s needs
7	Network with peers to develop ongoing learning and an evidence based knowledge for practice

The importance of practice nurses appraising the culture of the general practice in which they work as a first step in implementing a change to their practice to include cervical screening and well women’s health checks is a strong theme. A comparative, critical realist review of the development of practice nursing in the United Kingdom, New Zealand and Australia [11] argues that the culture of general practice in Australia has been slow to embrace a broader role for nurses and that this is largely related to the funding structures and business models of general practice which are often owned by general practitioners. As identified in the background to this study, recent changes from task based compensation for practice nurses’ work, to a more inclusive PNIP payment to support the employment of practice nurses have been introduced, however these changes are yet to be tested against the role of the PN.

Participants in this study report that identifying goals for their professional development that mesh with the general practice’s goals to increase service provision is an achievable strategy that promotes the establishment of nurse led cervical screening and well women’s health services. These findings contrast with earlier studies by Mills and Fitzgerald [3,4] and Jasiak and Passmore [2] that report GP resistance to practice nurses establishing new models of cervical screening and well women’s health services. In particular, Mills and Fitzgerald [3,4] identify female GPs as a barrier to PNs provision of cervical screening services. More recently however, Peters [12] suggests clients’ preference for a female providing this service has led to more male GPs relinquishing clients to their counterparts, the implications of which for female GPs’ workloads has motivated their support of PNs expanding their role. Continuing the theme of reducing GP’s workload through diversifying service provision, a general practice in a large provincial Queensland city has trialled the introduction of cervical screening by practice nurses [13] with a resultant 14.5% increase over a two year period in the overall number of cervical screens being undertaken in this practice [13].

In an investigation of women’s views of registered nurses as ‘Pap smear’ providers Christie et al. [1] found high levels of acceptance of “a female health professional [providing] a Pap smear” [1] (p.166) which agrees with findings from this study. These authors go on to argue that clients are less likely to accept cervical screening conducted by male PNs, citing personal comfort and the sensitivity of female clinicians as influencing factors. Supporting the position that clients prefer a female clinician to provide cervical screening services, Peters [12] concludes that when deciding on where to access routine health screening services such as ‘Pap smears’ and mammograms, women seek women-friendly and women-centred services. An added dimension from this study is that in many instances clients choose to have a PN provide cervical screening and well women’s health checks in preference to a GP, however it is unclear if this preference can also be attributed gender.

Findings from this study indicate a key enabler of PNs expanding their role is adequate resourcing. Resourcing includes elements of infrastructure such as space and equipment, in addition to clinical, policy and administrative support. The importance of resources such as space and time in PN’s exercising the full scope of their role is also identified in two other studies investigating the work of Australian practice nurses [14-16], including those practising in the speciality area of mental health.

Information systems are also an important resource for PNs in their role as well women’s health care providers. Currently in most states and territories of Australia cervical smears collected by PNs are reported using the Medicare Provider Number of the GP supervising their practice. The exception to this method of reporting is Victoria where aggregate data produced by the Victorian Cytology service demonstrates the contribution nurses make in reaching screening targets for cervical screening [17]. As a result of PNs not having their own Medicare Provider Number there is no capacity to determine who provided the service if the GP is also collecting cervical smears. In the document *Making Quality Visible*, it was identified that this lack of recognition of the nurse’s role had potential medico-legal implications as the cytology laboratory is unable to identify the actual provider [18]. This also has an impact on quality from two perspectives:

1. Firstly, the PN is not provided with a quality report (from the laboratory or Pap Smear Register) that is specific to the cervical smears they have collected and is therefore unable to determine the quality of the smears of these smears. In the absence of accurate quality reports, cervical smear providers are not able to determine their ongoing competency.

2. Secondly, state and territory programs are unable to accurately identify the number of RN cervical smear providers in their respective jurisdictions, which makes it difficult to effectively address workforce needs through training and communication strategies.

While the categorical results presented in this research report are rigorous when appraised using measures of quality including originality, resonance and usefulness [9,10] they are limited in their generalisability due to small numbers of participants who were not selected as a representative sample of all PNs. Further research, which includes GPs’ and clients’ perspectives of PN led cervical screening and well women’s clinics would allow for a clearer understanding of the process of implementing a nurse led clinic in general practice. Additionally, research that investigates the impact of recent funding changes for PN provided cervical screening and well women’s health checks is vital to ensure the continuation and strengthening of this part of their role.

## Conclusions

Previous government funding initiatives have influenced the role expansion of Australian PN’s role to include cervical screening and well women’s health services. Whilst Medicare data demonstrates an increase in the number of cervical screening and well women’s health care services provided by PNs over time, recent funding changes will limit service planners’ ability to continue monitoring this service because of the abolition of specific item numbers. Contributing to the difficulty of tracking PN activity in this area are state by state inconsistencies regarding unique cytology identifiers.

Findings demonstrate four enablers to successfully implementing a change in PNs practice to include cervical screening and well women’s health care services: GPs being willing to relinquish the role of cervical screener and well women’s health service provider; PNs being willing to expand their role to include cervical screening and well women’s health services; clients preferring a female practice nurse to meet their cervical screening and well women’s health needs; and the presence of a culture that fosters interprofessional teamwork. Of these four enablers, the manner in which clients’ preference for a female well women’s health care provider has influenced the culture of general practice, particularly in relation to the expanding the roles of PNs and improving collaborative practice with GPs is most important and demonstrates the burgening influence of consumers on the primary care landscape in Australia.

## Competing interests

The authors declare that they have no competing interests.

## Authors' contributions

JM, LC, EG, MK and CH made substantial contibutions to conception and design. JCS acquired the data, and together with JM undertook the majority of analysis. JM, JCS, LC, EG, MK and CH all contributed to the final analysis of the data. JM and JCS drafted the manuscript, while LC, EG, MK and CH revised it critically for important intellectual content. Each author has provided final approval of this version of the manscript to be published.

## Pre-publication history

The pre-publication history for this paper can be accessed here:

http://www.biomedcentral.com/1472-6955/11/23/prepub
